# 895. Clap Out: A Strategy to Increase Staff Engagement, Decrease Turnover, and Support Burn Patients

**DOI:** 10.1093/jbcr/irag033.391

**Published:** 2026-04-06

**Authors:** Tova Myers, Pamela Chris Linscott, Amy Knupp, Nicole P Bernal

**Affiliations:** The Ohio State Comprehensive Burn Center, Columbus, OH; The Ohio State Comprehensive Burn Center, Columbus, OH; The Ohio State University Wexner Medical Center, Columbus, OH; The Ohio State Comprehensive Burn Center, Columbus, OH

## Abstract

**Introduction:**

Nurse turnover remains a critical issue in healthcare contributing to staffing shortages, increased costs, and compromised patient care. The care of the burn patient involves significant levels of mental, emotional, and physical stress. Strategies focused on celebrating patient outcomes have the potential to build a resilient and positive unit culture. This initiative aims to build a culture of engagement, satisfaction, and resilience, with the goal of achieving and maintaining zero nurse turnover in an academic medical center.

**Methods:**

As hallmark of our unit culture, our staff have embraced the tradition of the “clap out,” which is a celebration for burn patients upon their discharge. As part of the “clap out,” the entire healthcare team is invited to line the hallway upon discharge and clap and cheer while the patient is wheeled out of the unit. This is organized with nursing unit leadership, case management and social work as part of the discharge process.

**Results:**

The common elements of patients that receive a “clap out” are a larger total body surface area burned, amputations or new physical impairments, or prolonged hospital course. This past fiscal year our burn unit had a 0% turnover rate among nursing staff. The “claps outs” help boost team morale and patient satisfaction. Staff feedback has expressed pride for the patients and families during these moments. This process provides staff with a safe space to witness vulnerability and the celebration of each individual’s contribution to the patient’s recovery.

**Conclusions:**

In a time where nursing shortage and burn out is a challenge, we have developed a fluid process celebrating our patients and their burn recovery journey while also celebrating our staff. The benefits of the ‘clap out” include staff engagement, improved patient experience, reinforced the mission of the unit, and allows visibility of the team through the hospital system.

**Applicability of Research to Practice:**

Retention data and anecdotal feedback from our unit’s initiative suggest that this celebration could be successfully adapted in other healthcare settings to promote workforce stability and high- quality care.

